# Sustainable healthy diet modeling for a plant-based dietary transitioning in the United States

**DOI:** 10.1038/s41538-023-00239-6

**Published:** 2023-11-28

**Authors:** Raphael Aidoo, Vincent Abe-Inge, Ebenezer M. Kwofie, Jamie I. Baum, Stan Kubow

**Affiliations:** 1 Department of Bioresource Engineering, McGill University (Macdonald Campus), 21111 Lakeshore, Ste-Anne-de-Bellevue, Québec, H9X 3V9 Canada; 2 Center for Human Nutrition, University of Arkansas System Division of Agriculture, Fayetteville, AR 72704 USA; 3 Department of Food Science, University of Arkansas, Fayetteville, AR 72704 USA; 4 School of Human Nutrition, McGill University, 21111 Lakeshore, Ste-Anne-de-Bellevue, Québec, H9X 3V9 Canada

**Keywords:** Environmental sciences, Agriculture, Health humanities

## Abstract

The potential environmental and nutritional benefits of plant-based dietary shifts require thorough investigation to outline suitable routes to achieve these benefits. Whereas dietary consumption is usually in composite forms, sustainable healthy diet assessments have not adequately addressed composite diets. In this study, we build on available data in the Food4HealthyLife calculator to develop 3 dietary concepts (M) containing 24 model composite diet scenarios (S) assessed for their environmental and nutritional performances. The Health Nutritional Index (HENI) and Food Compass scoring systems were used for nutritional quality profiling and estimates of environmental impact were derived from previously reported midpoint impact values for foods listed in the What We Eat in America database. The diets were ranked using the Kruskal‒Wallis nonparametric test, and a dual-scale data chart was employed for a trade-off analysis to identify the optimal composite diet scenario. The results showcased a distinct variation in ranks for each scenario on the environment and nutrition scales, describing an inherent nonlinear relationship between environmental and nutritional performances. However, trade-off analysis revealed a diet with 10% legumes, 0.11% red meat, 0.28% processed meat and 2.81% white meat could reduce global warming by 54.72% while yielding a diet quality of 74.13 on the Food Compass Scoring system. These observations provide an interesting forecast of the benefits of transitioning to an optimal plant- and animal-based dieting pattern, which advances global nutritional needs and environmental stewardship among consumers.

## Introduction

Plant-based diets (PBDs) are a growing trend in Western diets^1,2^ due to their established benefits on human health and the environment^3^. PBDs are diets that originate mainly from plant sources with no or small quantities of animal products, as described by World Health Organization^4^. The World Health Organization (WHO) further described PBDs to include mainly vegetarian diets such as vegan, pesco-vegetarian (pescatarian), ovo-vegetarian, lacto-ovo-vegetarian and lacto-vegetarian diets. However, in the work of Craig, et al.^2^, PBDs are defined as exclusively vegan and lacto-ovo-vegetarian diets, meaning, diets from plant origin with or without eggs and dairy products. These descriptions conflict with the description by Ostfeld^5^, who defined PBDs as minimally processed plant materials including spices and herbs and excluding all animal products. PBDs have been defined in different ways, probably influenced by the consideration of social, cultural, and agricultural variations of food systems in various geographic settings according to Horgan, et al.,^6^ Fanzo, et al.^7^. In this study, our description of PBDs follows the definition by World Health Organization^4^.

PBDs primarily contain plant materials such as fruits, vegetables, grains, legumes, nuts, and seeds, either unprocessed, minimally processed, or ultra-processed forms. Both minimally and ultra-processed forms exist, including processed plant-based alternative foods such as meat, milk, cheese, fish, and egg alternatives^8^. Ultra-processing compromises the health and sustainability benefits of plant-based foods^8^. For instance, ultra-processed foods are associated with higher sugar intake, which may confer detriments to human health^9^. In addition, ultra-processing often involves higher energy use and emissions into the environment. Nonetheless, plant-based foods are generally more sustainable with co-benefits of lowering human health risks and improving environmental health compared to animal-based foods^2^.

The shift toward plant-based diets is necessitated mainly by climate action strategies to reduce global warming through the reduction of greenhouse gas emissions^10^. Global food production emits approximately 17 billion metric tons of greenhouse gases, representing about 17–32% of global emissions^11–13^. Of this, meat production alone contributes approximately 57%, while plant-based food production contributes approximately 29%^12,13^. In the Canadian context, plant-based diets have been reported to account for a lesser portion (25%) of the carbon footprint of self-selected diets^14^. These findings support earlier reports that indicated that a shift toward plant-based foods would result in a lower environmental depletion caused by our food systems^15–17^.

In addition to their environmental benefits, transitioning to a plant-based diet is associated with lower risks of diet-related noncommunicable chronic diseases (NCDs)^2,18–22^. Approximately 80% of the total deaths in North America in 2016, including the US, were attributed to NCDs^23^. Prevalent among these NCDs are cardiovascular diseases, cancer, chronic respiratory diseases, and type 2 diabetes. Cardiovascular diseases (CVDs) have been reported as the leading cause of death among all NCDs in the US and worldwide^24,25^. Among the CVD risk factors are hypertension, obesity, hyperglycemia, and hyperlipidemia. The consumption of plant-based foods (mainly fruits, vegetables, pulses, nuts, seeds, and whole grains) has been found to be a mitigative mechanism for these risk factors in addition to lowering the risks of cancer and type 2 diabetes^19–22^.

The shift toward plant-based foods has been gradual yet significant, especially in Western countries^26^. For instance, Alae-Carew, et al.^1^ reported an increase of almost 96% in the proportion of individuals who consumed plant-based alternative foods in the UK over an 11-year period (from 2008 to 2019). In a similar timeframe (2009–2020), the proportion of adults in the United States who practice vegetarianism increased from 3% to 6%, indicating a 100% increase^27,28^. These trends confirm the estimation that the proportion of vegetarians in many countries is generally less than 10%^2^. This trend is, however, at great variance with India, where approximately 23–37% of the population is vegetarian^29^.

There has been an increase in the number of completely vegan restaurants in the US and Canada from 55 in 1993 to 970 in 2022, which translates into an increase by 1663.64%, indicating a more rapid growth rate compared to the corresponding 31.3% increase in population^30^. In addition, there are several other restaurants where vegetarian meals are sold. The ‘Meatless Monday’ campaign aimed at avoiding meat consumption for at least one day every week has been extended to all public schools in New York City^31^. In 2018, Berkeley in California passed a requirement dubbed “Vegan Monday” into a law that requires all city-owned and city-managed facilities and programs to provide vegan meals only on Mondays^32^. Other reports also indicate an expansion of vegetarian diets in establishments such as prisons, hospitals, schools, airlines, employee canteens and nursing homes^32–34^. Additionally, the 2020–2025 Dietary Guidelines for Americans (DGA) encourage the consumption of more fruits, vegetables, nuts, whole grains, and beans among all Americans aged at least one year^35^.

The plant-based foods market has been projected to hit US$162 billion by 2030^36^. The global market for dairy alternatives alone has been projected to hit US$25 billion by 2026^37^. The most common among these plant-based foods include plant-based yogurt, sausage, patties, cheese alternatives, creamers, and hot dogs^38,39^. Consequently, there have been decreases in the sales of meat (5%) in 2015 and dairy milk (15.8%) in 2018, as reported by Aschemann-Witzel, et al.^40^ and Mintel Reports^41^, respectively. This growth reflects the growing adoption of plant-based dietary patterns among consumers. A plurality (43%) of plant-based food consumers purchase milk alternatives^42^^,^ with lactose intolerance, curiosity, or mere preferences of these consumers being the major driving forces of their choices. More recently, increasing consumer awareness and concerns about the environmental burdens of food choices has also been a substantive but gradual transition to plant-based alternatives. Clearly, the dual sustainability health and environmental benefits of plant-based diets are not the sole drivers but part of a broader spectrum of health, social, economic, and environmental actors driving a shift to sustainable healthy dieting. Regardless of the consumers’ motivation, this shift is sustainable; nonetheless, the diversity of choice drivers pinpoints the need to engage interdisciplinarity and radical collaborations in enhancing this transition to sustainable dietary choices.

The impact of a transition to plant-based diets on diet quality, cultural identity, social behavior, nutritional, environmental and health performance requires a thorough investigation to identify and address possible challenges to adopting PBDs. Previous work has highlighted notable gaps that require further investigation. Fadnes, et al.^43^, who developed the Food4HealthyLife calculator, outlined optimal and feasible diet scenarios for an improved life expectancy. In this study, optimal scenarios (dominated by plant-based foods) are identified as the best choice of diet for achieving maximum life-year gains; however, due to varying geographic discrepancies in the food system, feasible diet scenarios were recommended as better substitutes for optimal scenarios. Despite the promising health benefits of these scenarios, their environmental impacts have not been investigated. Additionally, Stylianou, et al.^44^, who investigated the nutritional quality and environmental impacts of American diets, focused more on cooked single-food items and less on composite uncooked diets. Consequently, little attention has been given to the environmental quality and nutritional quality of composite diets.

Since foods are usually consumed in their composite forms, focusing attention on understanding the sustainability performance of diets from the composite perspective would augment and support dietary decisions through the presentation of more accurate and relatable sustainability information and tools. This could nudge consumers and other stakeholders into rethinking their food choices. Additionally, considering the complexities of fully eliminating animal-based foods, a complete transition to a plant-based dietary system could be more challenging than maintaining the current dietary system. It is, however, essential to transition to a pattern that could lead to improved nutritional and environmental health. Therefore, the Food4HealthyLife Calculator was employed in this study to develop model composite diet scenarios between the current and optimal diet scenarios with a focus on partially replacing animal-based meats with legumes, a prominent plant-based protein source, aiming to assess and project the nutritional quality, environmental impacts, and environmental-nutritional trade-offs of these alternative diet scenarios and ultimately identifying feasible sustainable dietary patterns. The methodologies by Stylianou, et al.^44^, Fadnes, et al.^43^ and Mozaffarian et al.^45^, among other nutritional databases, were adapted accordingly and deployed in the assessment of the focus sustainability indicators in this study.

## Results

### Nutritional Quality of Diets

The nutritional quality of a diet, food or nutrient encompasses its benefits and detriments on the health of the consumer. The nutritional quality of the model diets with regards to the enlisted factors was assessed as described in the methodology, and the results are presented herein. The nutritional quality of the diets consisted of their Food Compass and HENI scores and are presented in Table 1. Both scores increased as the scenarios approached the optimal model diet. Food compass scores ranged from 65.46 (current diet) to 81.73 (optimal diet). Approximately 91.7% (22 out of the 24 diet scenarios) had FCS above 71, whereas the current diet (S1M1/M2/M3) and S2M2 (0.83% legumes, 1.39% processed meat, 4.89 red meat and 4% white meat) diet models recorded 65.46 and 70.90, respectively. According to the Food Compass Score categorization, consumption of foods with scores below or equal to 30 should be minimized, foods scoring between 31 and 69 are to be consumed moderately and above or equal to 70 are recommended. The Food Compass Scores exhibited approximately 93.3% positive correlation with the HENI scores, which ranged from 60.21 min per 100 kcal in the current diet (S1M1/M2/M3) to 320.85 min per 100 kcal in the optimal diet. Diet scenarios with lower calorie densities yielded higher HENI scores. This is evidenced in the 91.8% negative correlation between the calorie contents and HENI scores.

**Table 1:** HENI and food compass scores of model diet scenarios.

Diet Scenario^a^	Scaled FCS Score	HENI Score
S1M1/M2/M3	65.46	60.21
S2M1	72.38	177.62
S2M2	70.90	177.01
S2M3	72.42	172.69
S3M1	72.48	176.45
S3M2	73.73	174.61
S3M3	72.81	172.55
S4M1	72.30	174.81
S4M2	73.08	173.52
S4M3	73.08	172.33
S5M1/M2/M3	71.79	168.06
S6M1	71.87	167.96
S6M2	73.38	169.84
S6M3	73.48	170.71
S7M1	73.77	181.73
S7M2	73.47	169.76
S7M3	73.44	170.50
S8M1	74.24	166.87
S8M2	73.46	169.65
S8M3	74.20	170.80
S9M1	73.16	171.55
S9M2	73.64	169.56
S9M3	74.13	169.21
S10M1/M2/M3	81.73	320.85

^a^See supplementary material (SD1-S3, SD1-S4, SD1–5, SD1-S6) for summary definitions of diet scenarios.

Despite the strong positive correlation (93.3%) between the HENI and Food Compass scores, each nutritional assessment criterion yielded different rankings of the intermediate diet scenarios, including the default feasible diet on the Food4HealthyLife calculator. For instance, the S7M1 (6.89% legumes, 1.39% processed meat, 1.44% red meat and 3.47% white meat) diet model obtained a better rank (2^nd^) under the HENI scoring system than under the Food Compass Scoring system, where it ranked 5^th^. In contrast, the S8M1 (7.56% legumes, 1.39% processed meat, 0.78% red meat and 3.47% white meat) diet model ranked better (2^nd^) under the Food Compass system than under the HENI system, where it ranked 23^rd^. Despite the varying ranks, the current (S1M1/M2/M3) and optimal (S10/M1/M2/M3) diet models obtained the same ranking under both nutritional quality profiling criteria.

The trend of nutritional quality observed in this study is similar to the findings of Mozaffarian, et al.^45^, who revealed that the Food Compass, NOVA, Health Star Rating and Nutri-Score nutrient profiling methods yielded dissimilar nutritional quality scores for American diets. Despite this conformity of our findings to previous studies^45^, the Food Compass Scores were more sensitive to the compositional changes in our model diets compared to the HENI scoring system. In the Food Compass, diet scenarios between the current diet and existing model feasible diet obtained an average FCS of 71.86, lower than the average score of 73.52 for diet scenarios between the existing model feasible and optimal diets. Thus, diet scenarios with compositional quantities closer to the current diet generally ranked lower than scenarios with compositional quantities closer to the optimal diet scenario under the Food Compass system. The opposite was observed in the HENI score ranking, where diet scenarios between the current diet and feasible diet recorded a higher average score (174.62 min) than diet scenarios between the feasible and optimal diet (170.68 min) scenarios. The variation in scores for diet scenarios between the current and feasible diet scenarios [standard deviation = 2.36 (FCS); standard deviation = 36.22 (HENI)] was generally wider than the variation (standard deviation = 0.62 (FCS); standard deviation = 3.70 (HENI)] in the diet scenarios between the feasible and optimal diet scenarios. The variation in trend is attributable to the difference in considerations for the two scoring systems. We could argue that if the need for nutrition security is a global call, then the variation in diet quality assessment method is not a fair option. Most likely, a concerted methodology or metrics for quality assessment would ensure that a quality diet in one region corresponds to a quality diet in another region. However, it would be rather unfair to make this idea dominate the nutrition arena due to the current dynamics in nutritional burdens across the world that dictate what a quality diet would be for a sect of people within a specified timeframe. A single global methodology would therefore work in an ideal world of common nutritional distribution.

### Domain contributions (%) to food compass scores

**Fig. 1:**
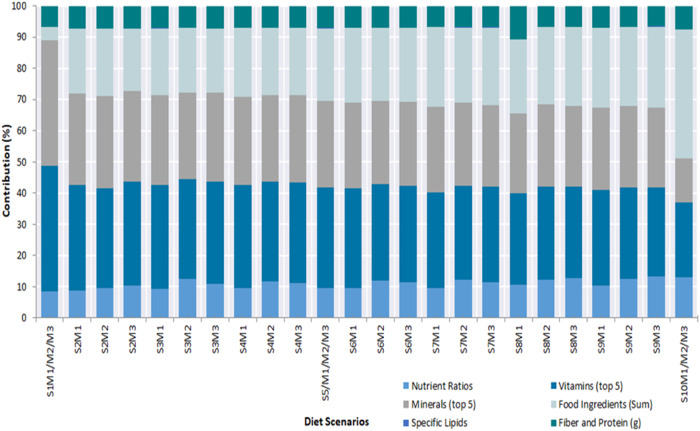
Domain contribution (%) to Food Compass Scores of the various diet scenarios.

Out of the seven (7) domains used in the Food Compass nutrient profiling of the diet scenarios, six (6) contributed to the obtained FCS, as shown in Fig. 1. On average, vitamins contributed the most (31.41%), whereas specific lipids contributed the least (0.051%). The nutrient ratio (27.36%), minerals (10.99%), food ingredients (23.19%) and protein and fiber (6.99%) domains also demonstrated significant contributions. Generally, the contribution of nutrient ratios and food ingredients increased as the diet scenarios approached the optimal diet. In contrast, the contribution of minerals and vitamins decreased, while the contribution of protein and fiber varied minimally (mean: 6.99, standard deviation: 0.23) as diet scenarios approached optimal. Consequently, approximately 80% of the Food Compass Score of the current diet scenario (S1M1/M2/M3) was contributed by the mineral (40.15%) and vitamin (40.24%) domains. These were reduced to 24.18% and 14.11%, respectively, in the optimal diet scenario. The food ingredients domain constituted 41.18% of the optimal diet Food Compass Score while contributing only 4.18% in the current diet scenario. Within the intermediate diet scenarios, food ingredients contributed the smallest (19.89%) to the S2M3 diet scenario (1.11% legumes, 2.5% processed meat, 4.89% red meat, 4.0% white meat) and the largest (26.09%) to S9M3 (10% legumes, 0.28% processed meats, 0.11% red meat and 2.81% white meat). Mineral and vitamin domains also contributed from 25.41% in S9M3 and 28.54% in S9M3 (10% legumes, 0.28% processed meats, 0.11% red meat and 2.81% white meat) to 29.46% in S2M2 (0.83% legumes, 1.39% processed meat, 4.89% red meat and 4% white meat) and 33.75% in S2M1 (0.67% legumes, 1.39% processed meat, 4.89% red meat and 3.47% white meat), respectively. The largest contribution of the protein and fiber domain was 7.61% in the optimal diet scenario (S1M1/M2/M3), with the lowest contribution of 6.63% in diet scenario S9M3 (10% legumes, 0.28% processed meats, 0.11% red meat and 2.81% white meat).

### Dietary risk components contribution (%) to HENI scores

**Fig. 2:**
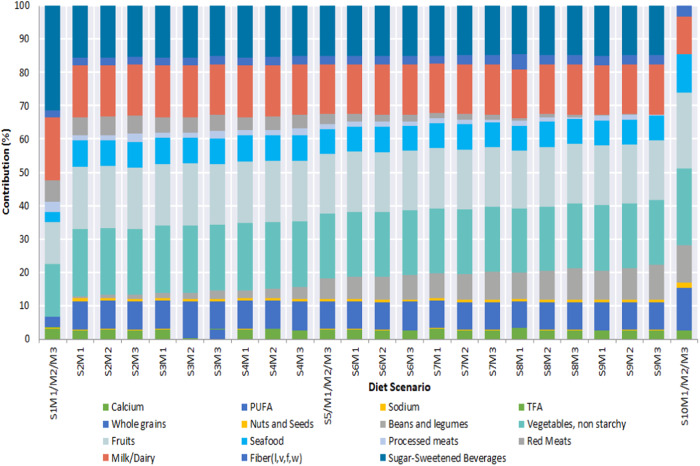
Contribution (%) of dietary risk components to HENI scores of the various diet scenarios.

Figure 2 presents the contribution of the dietary risk factors to the obtained HENI scores for the model diet scenarios. Vegetables, fruits, dairy, sugar-sweetened beverages, whole grains, seafood, beans/legumes, PUFAs, red meats and fiber are the main contributors to HENI scores; there were only minor contributions from sodium, calcium, TFA and nuts/seeds. On average, the greater contributors are vegetables (19.61%), fruits (18.09%), sugar-sweetened beverages (15.16%), milk/dairy (15.11%), whole grains (8.28%) and seafood (7.53%). The relative contributions of beans/legumes (5.59%), PUFAs (2.72%), red meats (2.74%), fiber (2.67%), processed meats (1.51%), seeds/nuts (0.75%), TFA (0.04%), calcium (0.16%) and sodium (0.04%) were also recorded. Hence, except for sugar-sweetened beverages, only minimal contributions were made from the harmful dietary risk factors, whereas beneficial dietary factors contributed largely. The percentage contribution increased from vegetables (15.75%), fruits (12.60%), seafood (3.15%), nuts/seeds (0%), beans/legumes (0%), fiber (1.98%) and whole grains (3.15%) to 22.83%, 22.83%, 11.42%, 1.43%, 11.42%, 3.22% and 12.84%, respectively, as the diet scenarios transitioned from the current diet to the optimal diet scenario. In contrast, the contribution decreased from sugar-sweetened beverages (31.50%), milk/dairy (18.90%), red meats (6.30%), processed meats (3.15%) and calcium (0.30%) to 0%, 11.42%, 0%, 0% and 0.04%, respectively. This trend of the percentage contribution of the dietary risk factors to the HENI scores reflects the compositional substitutions applied during the modeling of the diet scenarios.

### Environmental impacts of diets

**Fig. 3:**
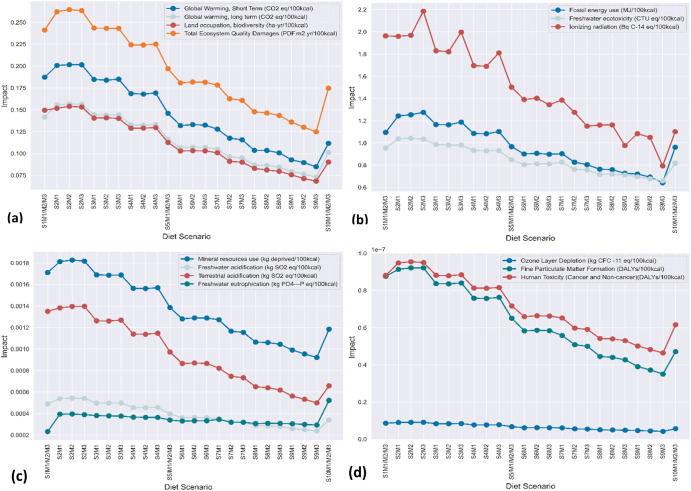
Estimated environmental impacts of diet scenarios. **a** Global warming short term, global warming long term, land occupation, total ecosystem quality damages. **b** Fossil energy use, freshwater ecotoxicity and ionizing radiation. **c** Mineral resource use, freshwater acidification, terrestrial acidification, and freshwater eutrophication. **d** Ozone layer depletion, fine particulate matter formation and human toxicity(cancer and non-cancer).

In recent times, the environmental impact of diets has been as important to consider as their nutritional and human health impact. As presented in Fig. 3, the estimated environmental impacts for the diet scenarios indicate greater environmental impacts resulted from fossil energy use, freshwater ecotoxicity and ionizing radiation, which ranged from 0.64–1.28 MJ, 0.66–1.04 CTU eq and 0.79–2.19 Bq C-14 eq per 100 kcal, respectively. These are followed by global warming (short term) (0.08–0.20 kgCO_2_ eq/100 kcal), global warming long term (0.07–0.16 kgCO_2_ eq/100 kcal), land occupation (0.07–0.15 ha-yr/100 kcal) and total ecosystem quality damage (0.12–0.26 PDF.m^2^. yr/100 kcal). The categories with the lowest impact were marine eutrophication, ozone layer depletion, photochemical oxidation, fine particulate matter formation, human toxicity (cancer and noncancer) and total human health damage. The diet scenarios also exhibited environmental impacts regarding mineral resource use, freshwater acidification, terrestrial acidification, freshwater eutrophication, and water use.

Generally, the environmental impacts of the diet scenarios decreased as the scenarios approached the S9M3 (10% legumes, 0.28% processed meats, 0.11% red meat and 2.81% white meat) diet scenario and increased afterwards in the optimal diet scenario (see Supplementary Data 2; Sheet SD2 – S2). For instance, transitioning from the current model diet/S1M1M2M3 (0% legumes, 2.78% processed meat, 5.56% red meat and 4.17% white meat) to the S9M3 (10% legumes, 0.28% processed meats, 0.11% red meat and 2.81% white meat) diet model reduced global warming by 54.72%. The S2M2 (0.83% legumes, 1.39% processed meat, 4.89% red meat and 4% white meat) diet scenario recorded the highest impact for all environmental impact indicators except for freshwater eutrophication, ionizing radiation, and fossil energy use. Similarly, the S2M1 (0.67% legumes, 1.39% processed meat, 4.89% red meat and 3.47% white meat) and S2M3 (1.11% legumes, 2.5% processed meat, 4.89% red meat and 4% white meat) diet scenarios yielded relatively higher impacts for all environmental impact indicators with the exceptions of ionizing radiation, freshwater eutrophication, and fossil energy use. An increase in global warming was observed in transitioning from the current diet scenario to the S2M1 (7.08%), S2M2 (7.59%) and S2M3 (7.56%) diet scenarios, where the authors propose a significantly increased intake of fruits, vegetables, whole grains, and fish in addition to the suggested changes in protein consumption (see Supplementary Data 1; Sheets SD1-S3, SD1-S4, and SD1-S5). This observation signaled an increased intake of sustainable foods, while maintaining the consumption rates of unsustainable diets could potentially be environmentally harmful. However, maintaining the consumption rates of healthy and sustainable foods while significantly transitioning from 0% legumes, 2.78% processed meats, 5.56% red meat and 4.17% white meat (current diet/S1M1M2M3) to 10% legumes, 0.28% processed meats, 0.11% red meat and 2.81% white meat (S9M3) significantly reduced global warming by 54.72%. Hence, the S9M3 diet scenario emerged as the most environmentally friendly diet scenario. S9M2 (8.89% legumes, 0.56% processed meat, 0.78% red meat and 2.97% white meat) emerged as the second most environmentally friendly diet scenario for all indicators except ionizing radiation, freshwater eutrophication, and water use. For instance, it demonstrated a potential reduction in global warming by 46.22% relative to the current diet scenario.

The current diet scenario recorded the second highest impacts but only for global warming in the short term, land occupation, mineral resource use, terrestrial acidification, ozone layer depletion and fine particulate matter formation. It, however, produced the least impact on freshwater eutrophication. Additionally, the optimal diet scenario, which contained the highest quantities of whole grains, nuts, legumes, vegetables, fruits, and seafood with the lowest quantities of red meat, processed meat, white meat, eggs, refined grains, dairy and sugar-sweetened beverages, recorded the highest impacts on freshwater eutrophication and water use, the third highest impact on marine eutrophication and the fourth highest impact on photochemical oxidation. It generally produced impacts higher than the impacts from the S9M3 (10% legumes, 0.28% processed meats, 0.11% red meat and 2.81% white meat) diet scenario, which contained higher amounts of red meat, processed meat, white meat, egg, refined grains, dairy and sugar-sweetened beverages than the optimal diet/S10M1/M2/M3 (11.11% legumes, 0% processed meat, 0% red meat and 2.78% white meat) scenario (see Supplementary Data 1; Sheets SD1-S3, SD1-S4 and SD1-S5).

The environmental impacts of all indicators except ozone layer depletion and ionizing radiation were lower in the current diet but increased in scenarios S2M1, S2M2 and S2M3, where the sum of quantities of whole grains, nuts, legumes, vegetables, fruits, and seafood began to increase, while the total sum of processed meat, red meat, white meat, dairy, eggs, refined grains, and sugar-sweetened beverages began to decrease. Buttressing this trend, the environmental impacts for diet scenarios between the current diet and the default feasible diet scenario/S5M1/M2/M3 (5.56% legumes, 1.39% processed meat, 2.78% red meat and 3.47% white meat) are higher compared to the impacts of diet scenarios between the default feasible and optimal diet scenarios for all environmental impact indicators. This trend is driven by the increasing partial replacement of the total sum of animal-based meats with legumes as the diet scenarios approached the optimal. This is due to the strong linkage of animal-based meats with greenhouse gas emissions^44,46,47^ and, consequently, global warming. Global warming in turn correlates well with most environmental indicators. In this study, both short- and long-term global warming demonstrated strong positive correlations (correlation coefficient > 0.80 at α = 0.01) with all environmental impact indicators with some nuances for water use and freshwater eutrophication. This is similar to the findings of Stylianou, et al.^44^. All other indicators apart from freshwater eutrophication and water use correlated similarly.

### Nutrition-environmental trade-offs

**Fig. 4:**
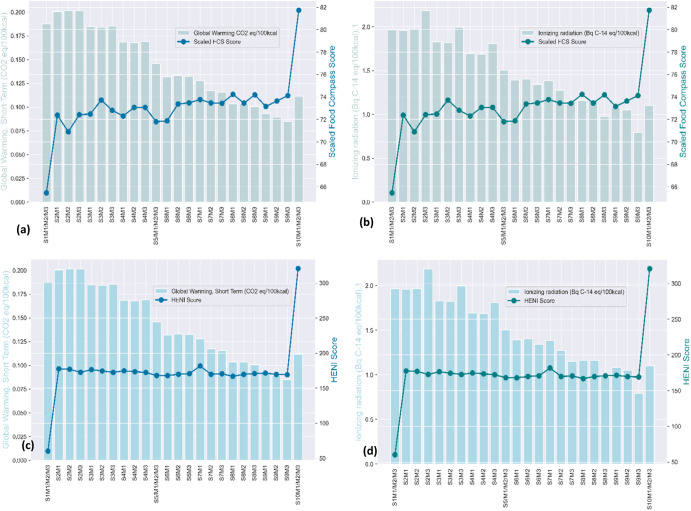
Nutritional-environmental trade-off analysis. **a** Scaled food compass score and global warming **b** Scaled food compass score and ionizing radiation **c** Global warming and HENI scores **d** Ionizing radiation and HENI scores.

**Fig. 5:**
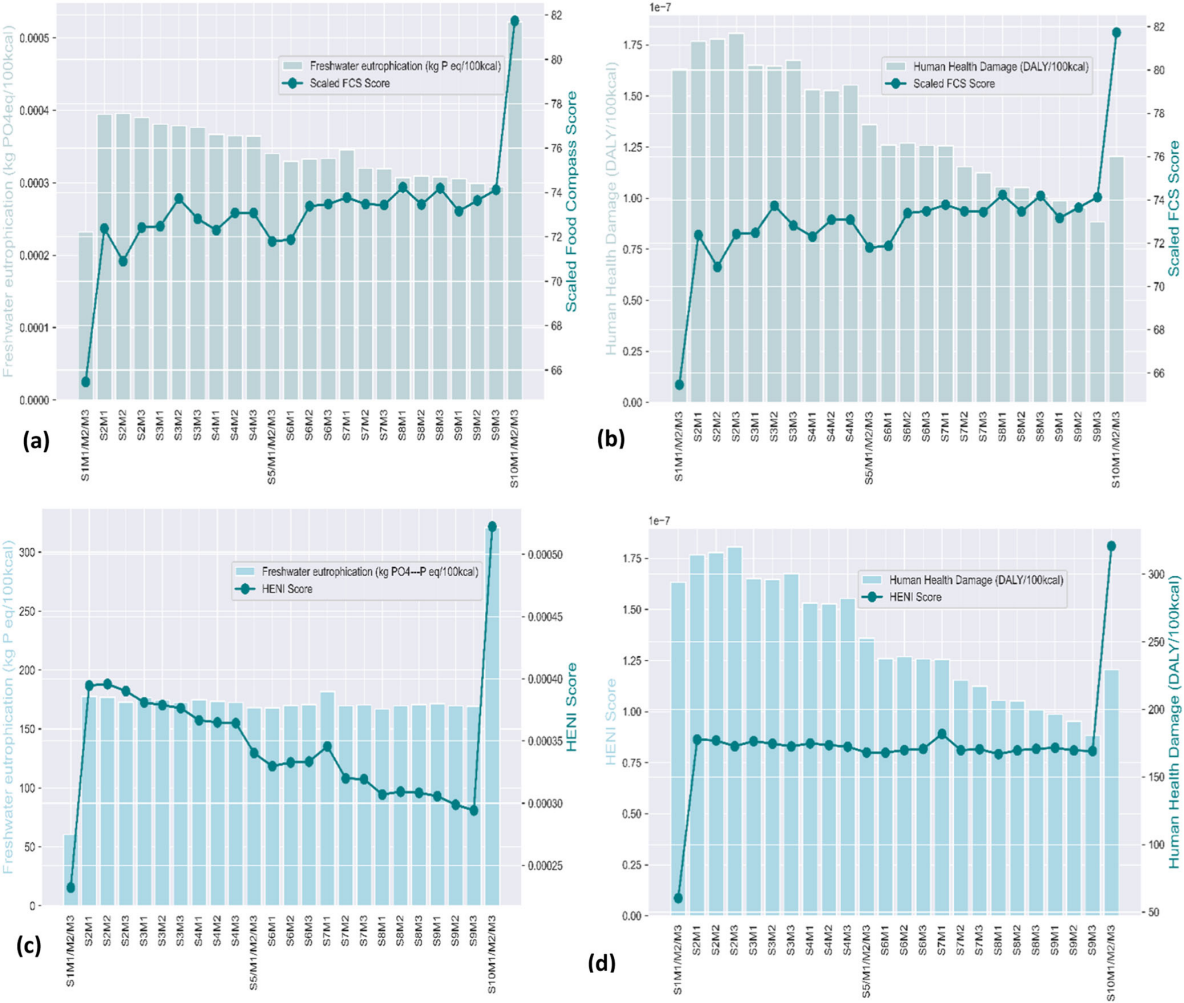
Nutritional-environmental trade-off analysis. **a** Scaled food compass score and freshwater eutrophication **b** Scaled food compass score and human health damage **c** Freshwater eutrophication and HENI scores **d** Human health damage and HENI scores.

Global warming exhibited a strong positive Pearson correlation (r^2^ > 0.80, α = 0.01) with all environmental impact indicators except water use and freshwater eutrophication. For this reason, the nutrition-environmental trade-off analysis was accomplished by utilizing each of the nutritional quality indicators with global warming in the short term, ionizing radiation, and freshwater eutrophication. The results obtained are presented in Figs. 4 and 5. Comparing the food compass scores with global warming impact, ionizing radiation and freshwater eutrophication showed a decreasing environmental impact with an increasing food compass score. A similar observation resulted from trade-off analysis using the HENI scores. However, a nuanced trend occurred in the optimal diet scenario, where in all the trade-off analysis scenarios, both the nutritional quality scores and environmental impacts increased. The increased quantities of fruits, vegetables, cereals, legumes, and nuts in this diet scenario could account for this trend. Thus, although the quantities of red and processed meat in the optimal diet scenario were zero, the significant increase in the quantities of fruits, vegetables, cereals, and legumes could account for the increased diet quality and environmental impact. Therefore, the environmental impacts reached their lowest in diet scenario S9M3 while yielding corresponding high nutritional quality scores. Apart from trade-off analysis using the food compass score and global warming, where the S2M2 diet scenario was the least sustainable diet scenario, the S2M3 diet scenario was the least sustainable in all trade-off scenarios involving global warming and ionizing radiation. When freshwater eutrophication was included in the analysis (Fig. 5), the S2M2 diet scenario became the least sustainable diet using the Food Compass scores, whereas S2M3 became the least sustainable diet. The least sustainable diet scenarios are described as diet scenarios that had low nutritional quality scores and high environmental impacts. Consequently, the S9M3 diet scenario, which had approximately 76% of its total animal-based meats replaced with legumes, is the most sustainable diet scenario and qualifies as the optimal diet since it resulted in both high nutritional quality and an approximately 55% reduction in global warming, which correlates well with most of the environmental indicators.

## Discussion

**Fig. 6:**
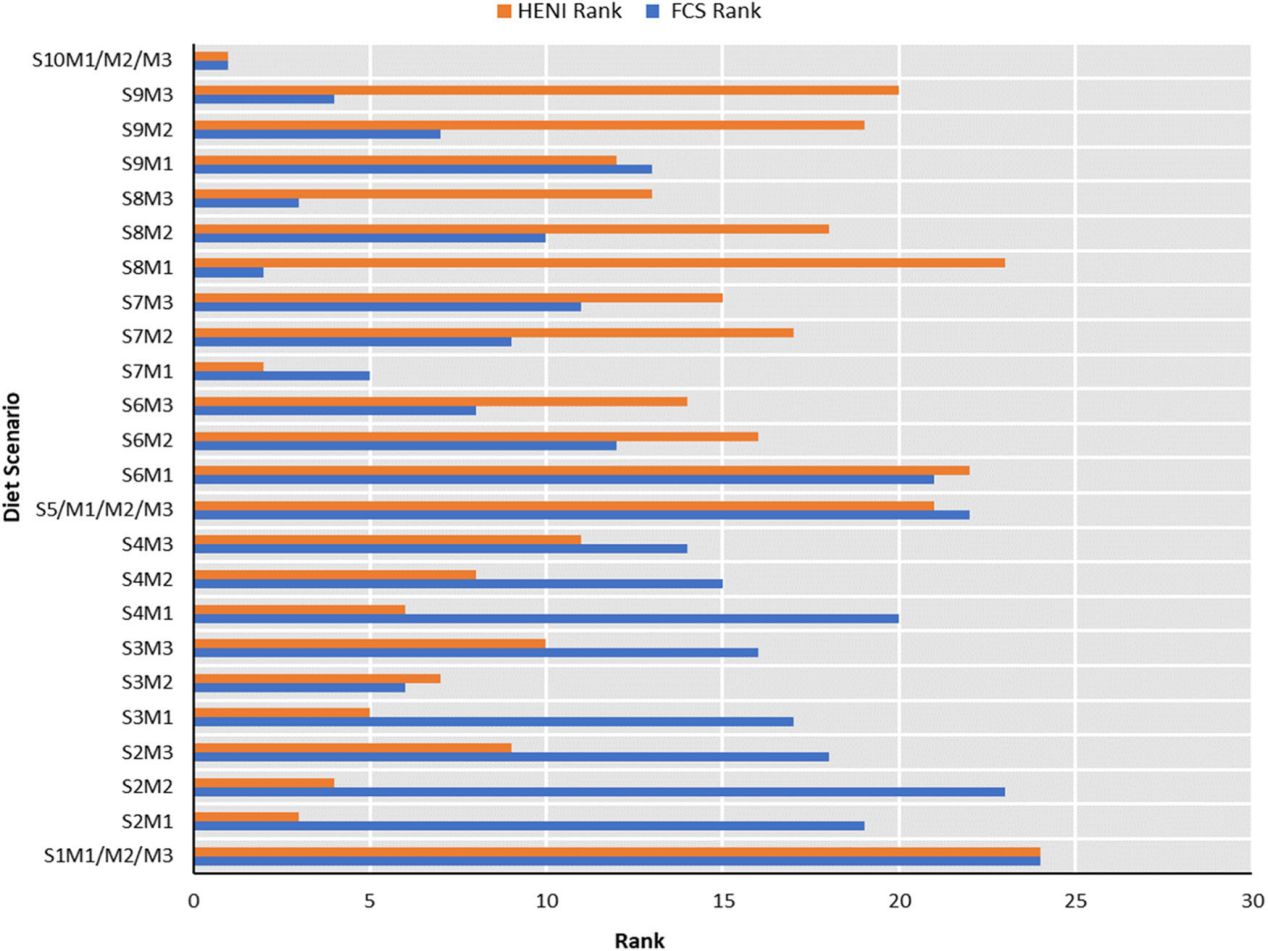
Rank of diets under the HENI and FCS characterization schemes (1= highest quality rank; 24 = lowest quality rank). See supplementary data 1 (Sheets SD1-S3, SD1-S4, SD1-S5) for details on composition of diet scenarios.

The S1M1/M2/M3 (current) diet scenario obtained the lowest Food Compass Score (65.46) due to its composition. It contains 100% animal-based proteins in comparison to the alternative diet scenarios, the lowest quantities of nuts, fruits, vegetables, whole grains, legumes, seafood, and the highest proportion of sugar-sweetened beverages. This combination should be nutritionally discouraged and is described as unhealthy due to its high composition of unhealthy ingredients (sugar-sweetened beverages, processed meats and red meats) and lower composition of healthy foods (whole grains, nuts, legumes, seafood, fruits and vegetables)^43–45,48,49^. Evidently, the food compass scores increased progressively when incremental proportions of the healthy food components were introduced in scenarios S2M1, S2M2 and S2M3 to the highest scores in the optimal diet scenario (S10M1/M2/M3). Despite this increase in the Food Compass Score, there was an associated decrease in the mineral and vitamin contents, as illustrated in Fig. 1 and Fig. 6. This could be attributed to the incremental partial replacement of the proportion of animal-based meats with legumes. Plant-based food substitutes have been reported to be deficient in calcium, vitamin D, vitamin B12 and iron^50–52^ hence the observation in this study. Regardless, the Food Compass scores increased due to the incremental change in the proportion of food ingredients as diet scenarios approached the optimal (Fig. 6). The variation in the diet ranking outcomes of the HENI and Food Compass criteria is similar to the findings of Mozaffarian et al.^45^ and associated with the robust diet calorie density dependence (r^2^ = −0.918) of the HENI scores. This linkage of HENI and calorie density was also reported by Stylianou, et al.^44^. To explain this, higher masses of low-calorie diets are required to attain the 100 kcal diet basis for the HENI scoring used in this study than for high-calorie diets. Consequently, lower-calorie foods attain higher HENI scores than higher-calorie foods. Intrinsically, the HENI scores were influenced by the increasing and decreasing contents of the healthy and unhealthy dietary risk components as the model diet scenarios transitioned from the current diet scenario to the optimal scenario, as illustrated in Fig. 2. Despite the variation in the ranking outcomes, both the Food Compass and HENI scores confirmed that our current dietary pattern is nutritionally inferior and that we must transition toward healthier dietary options.

In diet scenarios, S2M1, S2M2 and S2M3, the total quantity of healthy and environmentally friendly food groups increased (from the current diet), while the total quantities of high-risk (unhealthy) food groups decreased toward the optimal diet scenario. This dietary change initially demonstrated a 7.0–7.6% increase in the environmental impact estimates (at diet scenarios S2M1, S2M2 and S2M3) for all indicators except for ozone layer depletion and ionizing radiation, but these impacts decreased progressively to approximately 55% afterwards as the scenarios transitioned to the S9M3 diet scenario in which legumes constituted 10% whereas red, white, and processed meats constituted 0.11%, 2.81% and 0.28%, respectively. The lower environmental impact estimates from the current diet scenario (S1M1/M2/M3) for all indicators compared to diet scenarios S2M1, S2M2 and S2M3 (Fig. 3) imply the possibility of healthier diet options (mainly plant-based foods and seafood) exhibiting higher environmental damage than the unhealthier options depending on their type and quantities being consumed. This confirms previous reports that plant-based food groups are not always environmentally friendly and that the stigma surrounding animal-based foods may be an overgeneralization^44,50^. However, our results also reveal that maintaining the quantities of all other ingredients while further decreasing the total sum of animal-based meats through partial double-fold replacement with legumes caused a corresponding decrease in all environmental impact indicators. We therefore assert that although animal-based foods may not always be inferior to plant-based foods regarding sustainability, their reduction results in greater environmental benefits in almost all scenarios.

Separate evaluation of diets based on nutritional quality resulted in the optimal diet (S10/M1/M2/M3) obtaining the highest score, while the current diet (S1/M1/M2/M3) obtained the lowest score under both the Food Compass and the HENI criteria. In the environmental impact evaluation gauging the total human health damage, the optimal diet scenario ranks lower than eight diet scenarios, while the current diet ranks better than six diet scenarios. Partially (76%) replacing animal-based meat with legumes (S9M3) yielded milder environmental damage (global warming, ionizing radiation, and human health) than the optimal diet scenario where animal-based meats were 80% replaced, sugar-sweetened beverages were eliminated and consumption of fruits, legumes, vegetables, seafood, and whole grains was higher. Overall, the S9M3 (10% legumes, 0.28% processed meats, 0.11% red meat and 2.81% white meat) diet scenario simultaneously resulted in the highest environmental impact reduction (see Supplementary Data 2; Sheet SD2-S4) and a high nutritional quality score under both nutritional indicators. This trend of results proves that there is no correlation between nutritional quality and environmental emissions of diets^44^. This further implies that foods with higher life expectancy benefits could simultaneously be harmful depending on the quantities being produced and consumed. This reinforces the inadequacy of making dietary recommendations based on either only health benefits or only environmental benefits.

## Conclusion

The findings portray significant dynamics in the shift from animal to plant-based diet, attaching such shift with diversified trade-offs that need to be properly modeled to ensure optimal alignment with the environmental, nutrition, and health targets. In this study, increase in the percentage substitution of meat products with plant-based foods in the model composite diets reflected a non-linear variation in nutritional, environmental footprint, and health benefits, specifically for Food Compass Scores, HENI scores and environmental impacts of diets. This presents interesting perspectives about sustainable dietary modeling and formulation in the evolving plant-based dietary transition. Firstly, the insights understate that a sustainable transition does not solely revolve around increasing and reducing the quantities of plant and animal-based diets, respectively, but is entrenched typically in an augmented substitution modeling that renders significant environmental impact offset while providing optimal nutritional and health benefits. Thus, beyond interest in making this transition possible, properly designing a diet modeling technique that facilitates a more sustainable animal-based diet substitution should be prioritized. Secondly, the findings corroborate previous indications of no correlation between environmental impact and nutrition, suggesting that the quality of diets should be assessed and judged based on both their environmental and nutritional benefits. This supposes that a significant rapid dietary shift may not necessarily lead to the expected reduction in environmental and health damages caused by the current food systems unless the quantity, type, characteristics, and source of food are modeled as key variables in such a shift. For instance, while increment in plant-based composition in some of the composite diets rendered significant environmental impact offsets, the contrary was noted for the nutrition and health variables. Notably, the supposed sustainable plant-based dietary shift may have an increased potential to cause adverse environmental and human health damages without proper tracking and optimization of proposed interventions. However, overall, the study highlights a simultaneous decrease in animal-based foods such as red meat, white meat, and processed meat, while increasing plant-based food such as legumes to generally render desirable environmental nutrition co-benefits. In this vein, a regular composite diet with 10% legumes, 0.11% red meat, 0.28% processed meat and 2.81% white meat, would typically offset about 55% of global warming impacts while yielding a diet quality of 74.13 on the Food Compass Scoring system and redeeming about 169.21 min of daily adjusted life years relative to the current regular daily diet scenario for health people.

Although the study outlines a new easy-to-use approach for assessing the combined benefits of diets on human and environmental health, it comes with some limitations. The study relied on secondary midpoint environmental impact results for diets listed in the WWEIA database. Hence, the trends identified, and conclusions drawn from this study may not apply to food habits and choices in other regions of the world where the model may require the inclusion of different representative plant-based commodities. Additionally, data for phytochemical and carotenoid contents of our diet scenarios were not available and hence were excluded from the Food Compass scoring procedure, and other intrinsic factors like bioavailability were not included in this study. Again, the effect of cooking losses on the nutritional quality of our diet scenarios was not considered in this study because of the apparent variation in cooking methods across processors and households and how they impact nutrition differently. However, future studies could explore the possibility of simulating commercial and household cooked composite diets for real-time representation. Notwithstanding, the findings from this study present a reliable platform for policy and stakeholder decisions, implicating that nutritional, health, and environmental co-benefits could be achieved through sustainable dietary modeling.

### Methodology

**Fig. 7:**
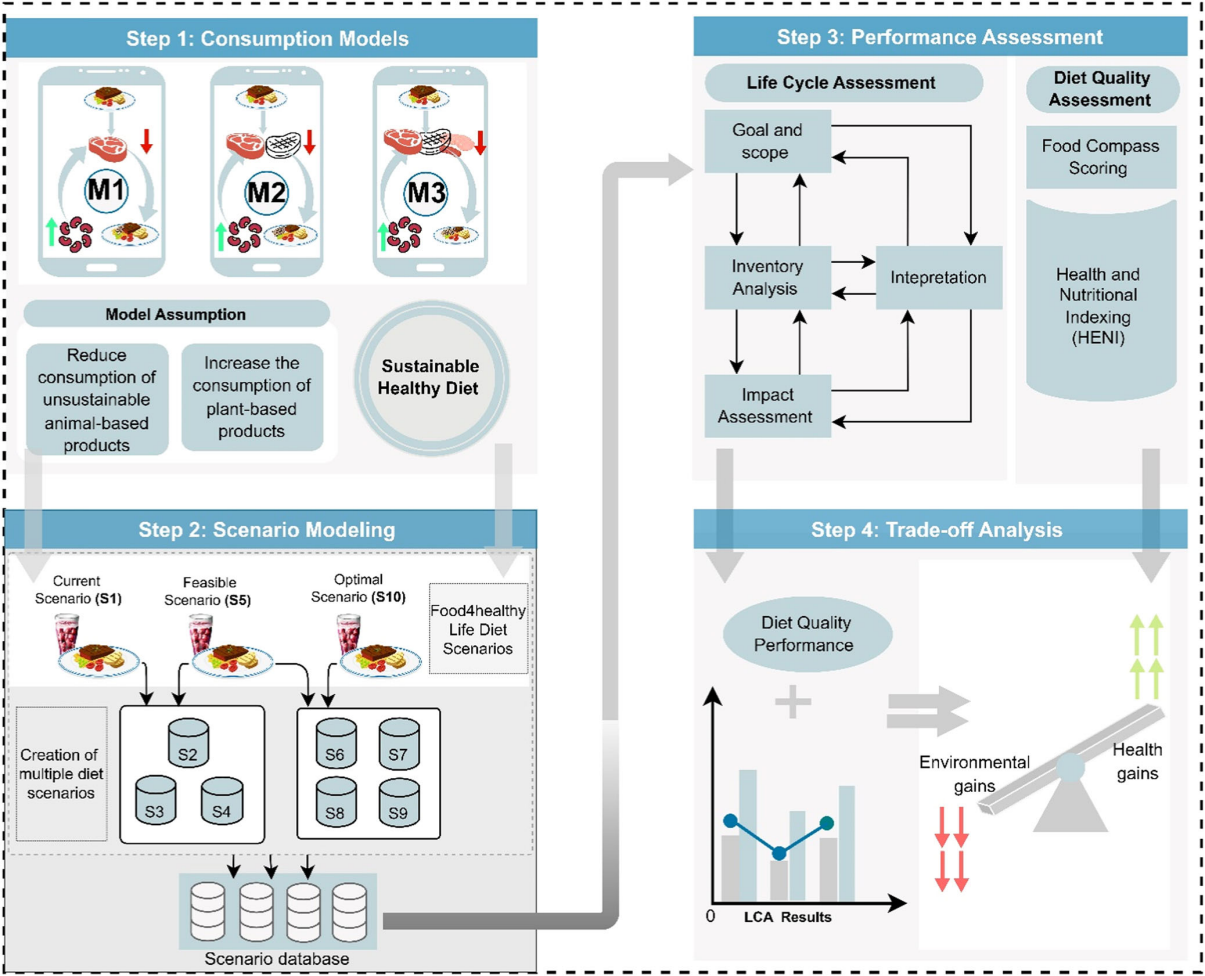
Summary of Methodological Framework. M = Model; S = Scenario.

The methodology is subsectioned into four steps, viz., the creation of the consumption models, development of diet scenarios, performance analysis including environmental impact and diet quality assessment, and an environmental nutrition tradeoff analysis. A graphical summary of the methodology is presented in Fig. 7.

### Consumption model and diet scenario design

Meat-based foods are linked with noncommunicable diseases and negative environmental impacts. The reduction in the consumption of meat, especially processed and red meat, has been revealed to be beneficial to both the planet and human health. In this study, model diets were developed based on these previous findings using the Food4HealthyLife calculator^43^. The Food4HealthyLife calculator was developed based on diet and life expectancy modeling: it defines current, feasible and optimal dietary patterns in relation to life expectancy. The current diet refers to the current dietary consumption pattern, which was found to significantly reduce life expectancy. The optimal diet refers to a hypothetical dietary pattern that was found to increase life expectancy but was not feasible (not applicable), whereas the feasible diet refers to the midpoint diet between the current and optimal diets and is applicable in relation to the available food supply patterns. Three diet consumption models (M1, M2, and M3) were developed, all of which targeted a partial replacement of meat with legumes. Consumption model 1 (M1) targeted a replacement of red meat only; consumption model 2 (M2) targeted red meat and white meat only; consumption model 3 (M3) targeted red meat, white meat, and processed meat (M3). For each consumption model, 7 diet scenarios (model diet) (S) were created in addition to the default current (S1), feasible (S5) and optimal (S10) diet scenarios described in the Food4HealthyLife calculator, summing up to 10 hypothetical diet scenarios (Supplementary Data 1; Sheets SD1-S3, SD1-S4, and SD1-S5). The 21 alternative diet scenarios were created using arithmetic mathematical equations developed by the authors based on the current, feasible, and optimal weights of the targeted food group(s) in the Food4HealthyLife calculator. The consumption models consisted of 14 food groups according to the categorizations in the Food4HealthyLife calculator, Food Compass, and Environmental Nutrition Food Groups. The food groups included whole grains, refined grains, nuts, legumes, fruits, fish, red meat, processed meats, dairy, eggs, white meat, added oils and sugar-sweetened beverages. For each food group, a representative food was selected on the premise of the most commonly available and consumed foods using the USDA Food Availability and Consumption Database as a guide. The total weight of each diet scenario amounted to 1.8 kg (the baseline total daily weight of the default current, feasible and optimal diets in Food4healthyLife Calculator^43^). In this scenario modeling, the impact of cooking and storage losses was excluded. Further information on the modeling procedure can be found in Supplementary Data 1 (Sheets SD1-S3, SD1-S4, SD1-S5).

### Nutritional quality assessment of model diets

The nutritional profile of the model diets was assessed following two (2) steps. First, the nutritional contents per 100 kcal of the model diet scenarios were determined using the FoodStruct online diet analysis system. Second, the Food Compass Scoring system^45^ was used to rate each model diet. The details of these steps are provided in the following sections: Nutrient Content Analysis of Diets and Food Compass Scoring of Diets.

### Nutrient content analysis of diets

The Diet Analysis menu of the FoodStruct online system was used to analyze the selected diets by providing the individual quantities of ingredients in each diet scenario. The contents of macronutrients, including carbohydrates, proteins, lipids, and fiber, and micronutrients, including 9 minerals and 12 vitamins, were determined. Other dietary components, such as specific lipids, including cholesterol, alpha-linolenic acid, eicosapentaenoic acid (EPA) and docosahexaenoic acid (DHA), were estimated from the nutrient analysis database. Further details are presented in the supplementary data 1 (Sheet SD1-S7).

### Food compass scoring (FCS) of diets

The nutrient profiles of the model diets were scored using the Food Compass Scoring Nutrient Profiling System developed by Mozaffarian, et al.^45^ with some slight modifications. Unlike other nutrient profiling systems, such as NOVA, Health Star Rating and Nutri-Score, Food Compass possesses the strengths of objectivity, universal applicability, consistent scoring attributes for all foods and a comparable unit of 100 kcal, warranting its consideration in our study. The nutrient contents of the diets were estimated per 100 kcal since the Food Compass compares food items per 100 kcal. Attributes such as iodine, trans-fats, medium-chain fatty acids, total flavonoids, total carotenoids, and processing as well as phytochemical domains were excluded from the attributes and domains used in this study. The processing domain could not be included since our diet models consisted mainly of uncooked ingredients. Additionally, the phytochemical domain was excluded because data were not readily available. Aside from this, we presumed a bearable effect of ignoring this domain since most attributes under this domain could be lost during household or industrial processing of the model diets. Hence, a total of 46 attributes and 7 domains were adapted from the Food Compass system for this study. Unlike in the Food Compass, red meats and processed meats were separate attributes in this study. Additionally, cutoff targets for food ingredients, originally quantified in cups and ounces in the Food Compass nutrient profiling system, were converted to grams prior to assigning the Food Compass scores. The final food compass score (FCS) was calculated as the sum of the average domain score and sum of scores for the food ingredients domain. The obtained Food Compass Scores were scaled to 100 for comprehensive comparability using Eq. (1).

1 $${\rm{Food\; Compass\; Score}}({\rm{FCS}})=100-\left(\frac{26.1-{original\; score}}{36.7}\right)* 99$$

### Health Nutritional Index (HENI) scoring of diets

The Health Nutritional Index is a health burden-based tool for assessing the human health impact (disability adjusted life years (DALYs)) of food groups and diets based on epidemiological evidence from the Global Burden of Disease studies^44,53^. Following the methodology described by Stylianou, et al.^44^, Stylianou^53^, HENI scores were determined per 100 kcal of the developed diet scenarios using the HENI factors for the 9 major food groups and 6 nutrients termed collectively as dietary risk factors by GBD 2017 Diet Collaborators (2019). The dietary risk factors and their corresponding HENI factors are shown in Table 2. The composition of food groups and nutrients in each diet in this study (see details in supplementary data 1, Sheet SD1-S9) were multiplied by their corresponding HENI factors. The obtained HENI in DALYs was converted to minutes using Eq. (2):

2 $${{HENI}}_{{diet}{scenario}}=-0.53\mathop{\sum}\limits_{r}{{HENI}\,{Factor}}_{r,{DALY}}\,\cdot\, {d}_{{diet}{scenario},r}\,$$

**Table 2:** Dietary risk factors and their corresponding HENI factors.

Dietary Risk	HENI Factor
Calcium	−5.1000
Polyunsaturated fatty acids	−0.6000
Sodium	13.9000
Trans fats	4.4000
Whole grains	−0.3400
Nuts and Seeds	−1.5000
Legumes	−0.2300
Vegetables	−0.0830
Fruits	−0.1800
Seafood	−81.000
Processed meats	0.8600
Red meats	0.0990
Milk	−0.0077
Fiber	−0.1900
Sugar-Sweetened Beverages	0.0660

Source: Stylianou et al.^42^.

### Estimation of environmental impacts of diet scenarios

The environmental impacts of the developed diet models were estimated using dietary environmental impacts reported by Stylianou, et al.^44^ for American diets. The study reported the average environmental emissions for 160 foods listed in the What We Eat in America (WWEIA) database. The environmental impacts included 18 environmental impact indicators comprising global warming (short term), global warming (long term), water use, ionizing radiation, mineral resources, freshwater ecotoxicity, ozone layer depletion, fine particulate matter formation, freshwater acidification, fossil energy use, marine eutrophication, land occupation, freshwater acidification, freshwater eutrophication, terrestrial acidification, human toxicity (cancer and noncancer), total ecosystem quality damage and total human health damage. For this study, the same environmental indicators were used. Representative foods in WWEIA were selected for the ingredients in the model diets of this study (Supplementary Data 1, Sheet SD-S1). The environmental impact of a given indicator for a given model diet was estimated by summing the environmental impact of each ingredient in the given diet. To elaborate further, the impact of a given environmental indicator for a given diet was calculated by (1) dividing the mean environmental impact of the given indicator by the reference amount customarily consumed (RACC), which resulted in a factor, and (2) multiplying the resultant factor by the weight of the given ingredient in the model diets created in this study. This resulted in the environmental impact for the given ingredient (3) summing up the impact for each ingredient in the given diet scenario. The summed impacts of a given environmental indicator for each ingredient equaled the environmental impact of the same indicator for the entire diet scenario (see details in supplementary data 2, Sheet SD2-S1).

### Nutritional-environmental trade-off analyses

The nutritional-environmental trade-off analyses were performed using dual-scale data charts. Both the HENI and Food Compass scores together with scores for short-term global warming, ionizing radiation, freshwater eutrophication, and total human health damage were the parameters used. The global warming scores correlated well (r^2^ > 0.8) with all the environmental impact indicators except freshwater eutrophication and water use. Due to this correlation, global warming was the main environmental impact indicator used in the trade-off analysis. Ionizing radiation was also used because its values were the highest among all the indicators, including having the highest impact values among all the indicators that affect human health and among indicators utilized for the environmental DALY calculations of products. Finally, the freshwater eutrophication impact was used since its values followed a pattern different from all other indicators and since it demonstrated no correlation with all other environmental indicators. Therefore, the trade-off analysis was performed using the Food Compass Scores with each of the selected indicators (global warming short term, ionizing radiation, and freshwater eutrophication). This trade-off analysis was repeated using the HENI scores.

### Statistical analysis

Pearson correlation analysis was carried out to assess the correlation between calories, nutritional quality indicators and environmental impact indicators of diet scenarios. The Kruskal‒Wallis nonparametric test was used to rank diet scenarios using the HENI scores, Food Compass scores and total human health damage. Statistical analyses and data visualization were carried out using Microsoft Excel 2016, Statistical Package for Social Sciences version 25 and Python 3.10.5 coupled with Jupyter Notebook v5.0.

### Supplementary information

Supplementary Data 1

Supplementary Data 2

nr-reporting-summary

## Data Availability

The authors declare that all datasets used and generated in this study are presented in the article and Supplementary Data 1 and 2.
